# Spinal Screening MRI Trends in Patients with Multiple Hereditary Exostoses: National Survey

**DOI:** 10.7759/cureus.6452

**Published:** 2019-12-23

**Authors:** Blake K Montgomery, Eli M Cahan, Steve Frick

**Affiliations:** 1 Orthopaedic Surgery, Stanford University, Palo Alto, USA; 2 Orthopaedics, Stanford University, Palo Alto, USA

**Keywords:** multiple hereditary osteochondromas, osteochondroma, spine, mri, screening, multiple hereditary exostoses

## Abstract

Background

Multiple hereditary exostoses (MHE) is a rare disease characterized by multiple osteochondromas. Osteochondromas growing into the spinal canal can produce devastating consequences, including permanent neurologic deficits and even death. Routine screening of the entire spinal canal with magnetic resonance imaging (MRI) is a controversial topic lacking a clear consensus or recommendation to guide decision-making. This study presents a case of an intracanal osteochondroma at C1 identified by routine screening and a survey describing current practices of MHE experts.

Methods

MHE experts were surveyed. Survey questions addressed multiple aspects of care, including the type of practice center, the volume of patients with MHE, and current screening practices.

Results

A total of 104 experts were contacted, with a total of 26 experts participating in the survey and 23 completing the entire survey. Seventy-two percent of respondents do not perform a routine MRI screen of the spine. For experts that routinely screen, screening is performed across a wide/variable age range (4-18 years).

Conclusion

Screening protocols for MHE patients to identify osteochondromas within the spinal canal has struggled to reach consensus due to the rarity of the disease. Recent literature provides conflicting advice for patients without neurological symptoms. Our study demonstrates that even experts who are leading the field demonstrate wide practice variability. Most respondents do not routinely perform screening spinal MRI. Due to the wide variability, a national guideline is needed to help guide physician and parental decision-making for patients with MHE. Our case illustrates the potential benefit of identifying an osteochondroma within the spinal canal at a location where further growth could have devastating neurological sequelae.

## Introduction

Multiple hereditary exostoses (MHE) is a rare autosomal dominant disease characterized by multiple osteochondromas [1]. Osteochondromas typically arise from the metaphysis of long bones, however, spinal osteochondromas may occur in up to 68% of patients [2-7]. The cervical spine is the most common location of osteochondromas within the spine, however, the thoracic and lumbar regions of the spine can harbor osteochondromas as well [5,8]. Osteochondromas invading the spinal canal (intracanal) occur in 4%-27% of patients and can lead to severe neurologic sequelae and even death [5,9-12].

Screening spinal MRI can be performed in asymptomatic patients with MHE to assess for spinal and intracanal osteochondromas. The utility of screening spinal MRI of the entire spine in patients with MHE is a debated topic without clear guidelines or recommendations. Isolated healthcare providers must decipher the conflicting body of evidence and make an independent decision regarding whether to perform a screening MRI for their patients with this rare disease. We sought to understand how experts are interpreting the controversial body of evidence. The aim of this study was to determine national trends in routine screening spinal MRI for patients with MHE. For context, we present an accompanying, illustrative case of a large C1 osteochondroma identified with routine screening MRI in an asymptomatic nine-year-old female.

## Materials and methods

Methods

A PubMed search using the keywords “multiple hereditary exostoses” was conducted to identify authors affiliated with Orthopaedic Surgery departments who have published on MHE in the last 10 years. These authors were considered experts. Experts were contacted via email and asked to participate in an online survey (Appendix). The survey was available from January 2019 to April 2019. The survey contained nine questions and addressed the type of practice center, the volume of patients with MHE, and current screening practices. Surveys were conducted, recorded, and analyzed on SurveyMonkey.

Illustrative case

A nine-year-old, right-hand dominant female with MHE (EXT1 mutation) presented to the clinic for the evaluation of progressive varus forearm and elbow deformity, decreasing the range of motion and pain. Forearm radiographs identified multiple osteochondromas as well as a dislocation of the radial head. Her pertinent exam findings included limited motion of her right forearm and elbow and a normal neurologic exam, including normal reflexes and gait. The family elected for surgery for her right forearm.

**Figure 1:**
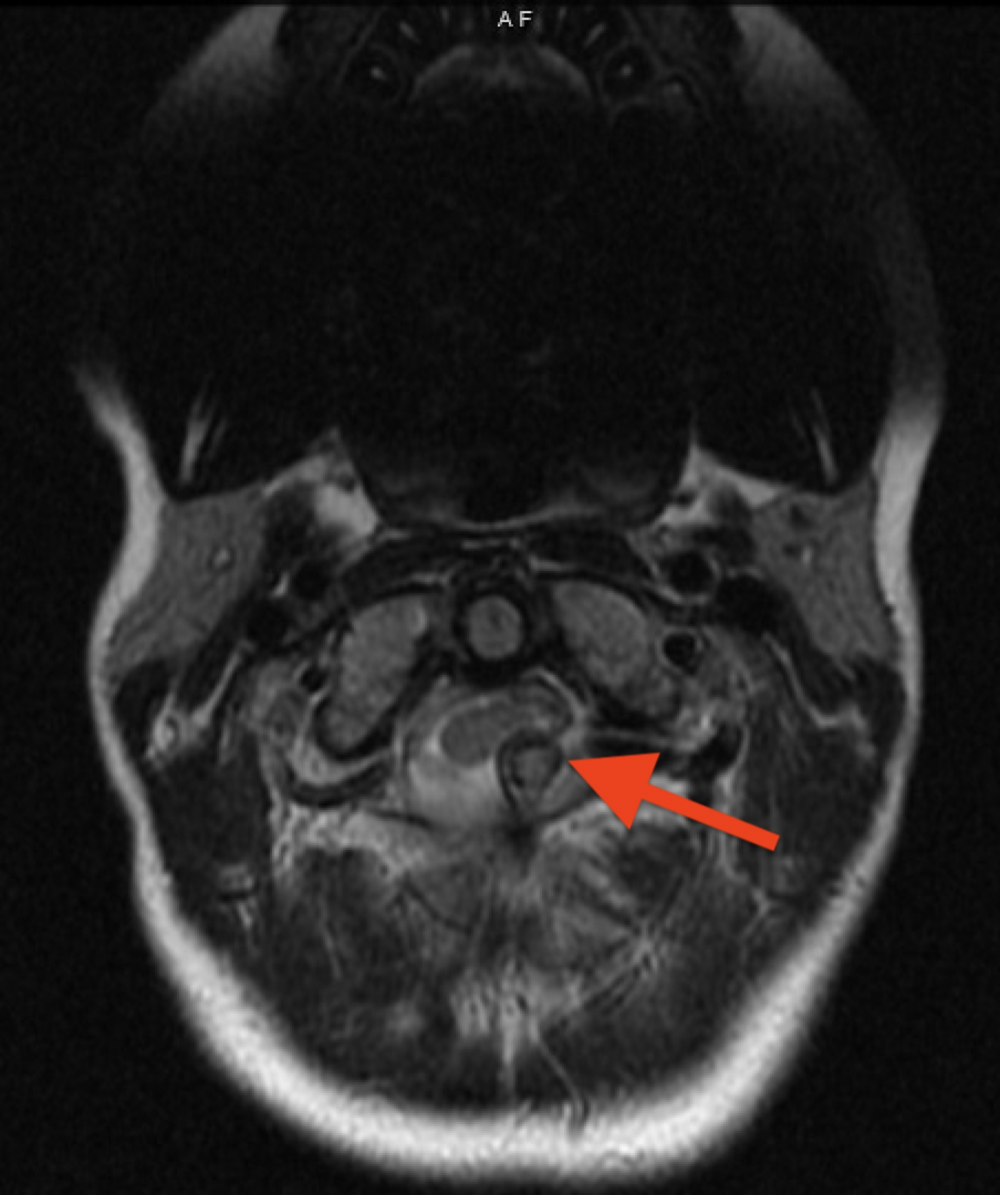
Cervical spine MRI axial view demonstrating an osteochondroma extending from the posterior arch of C1 and compressing the spinal cord

**Figure 2:**
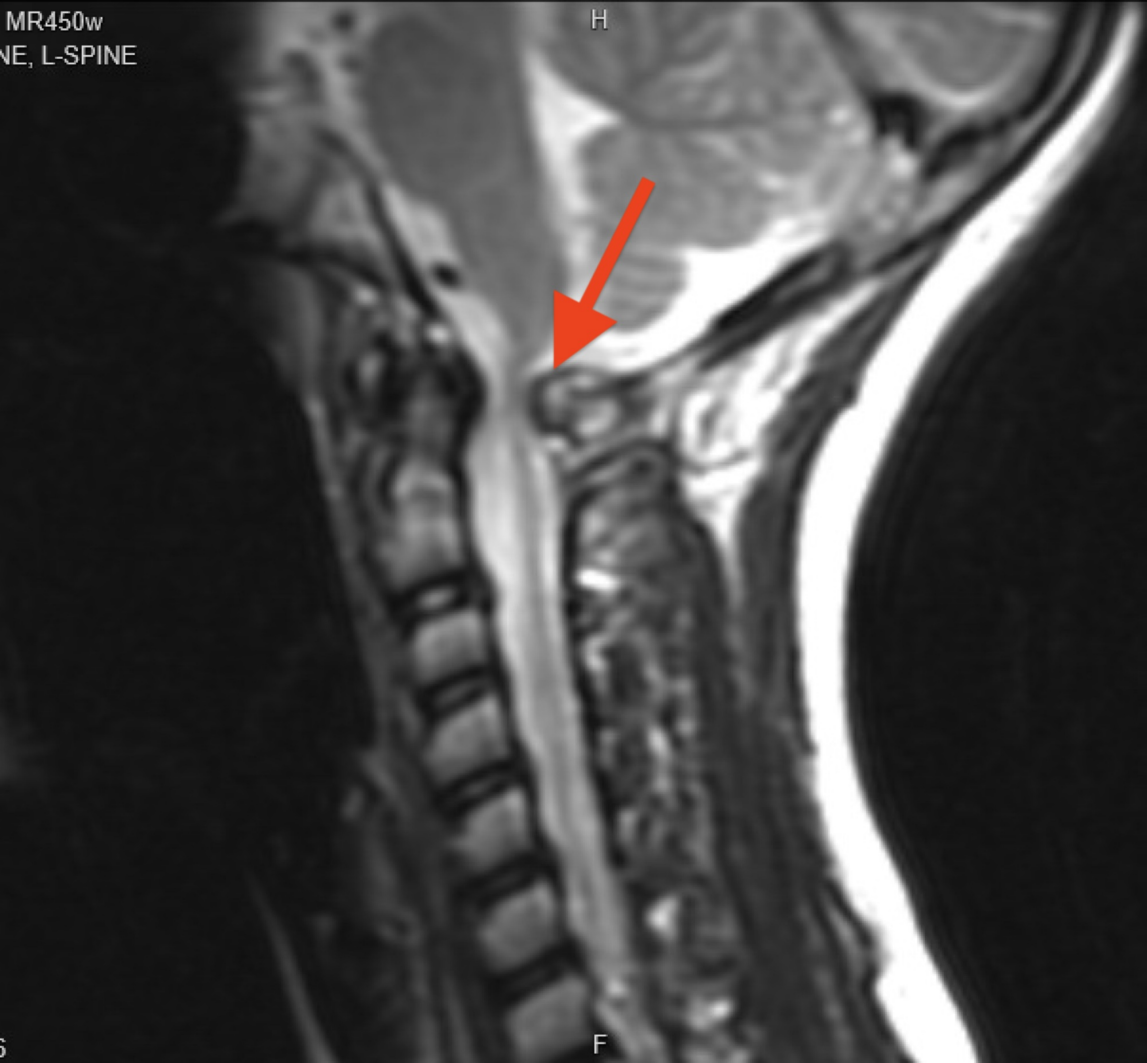
Cervical spine MRI sagittal view demonstrating an osteochondroma extending from the posterior arch of C1 and compressing the spinal cord

**Figure 3:**
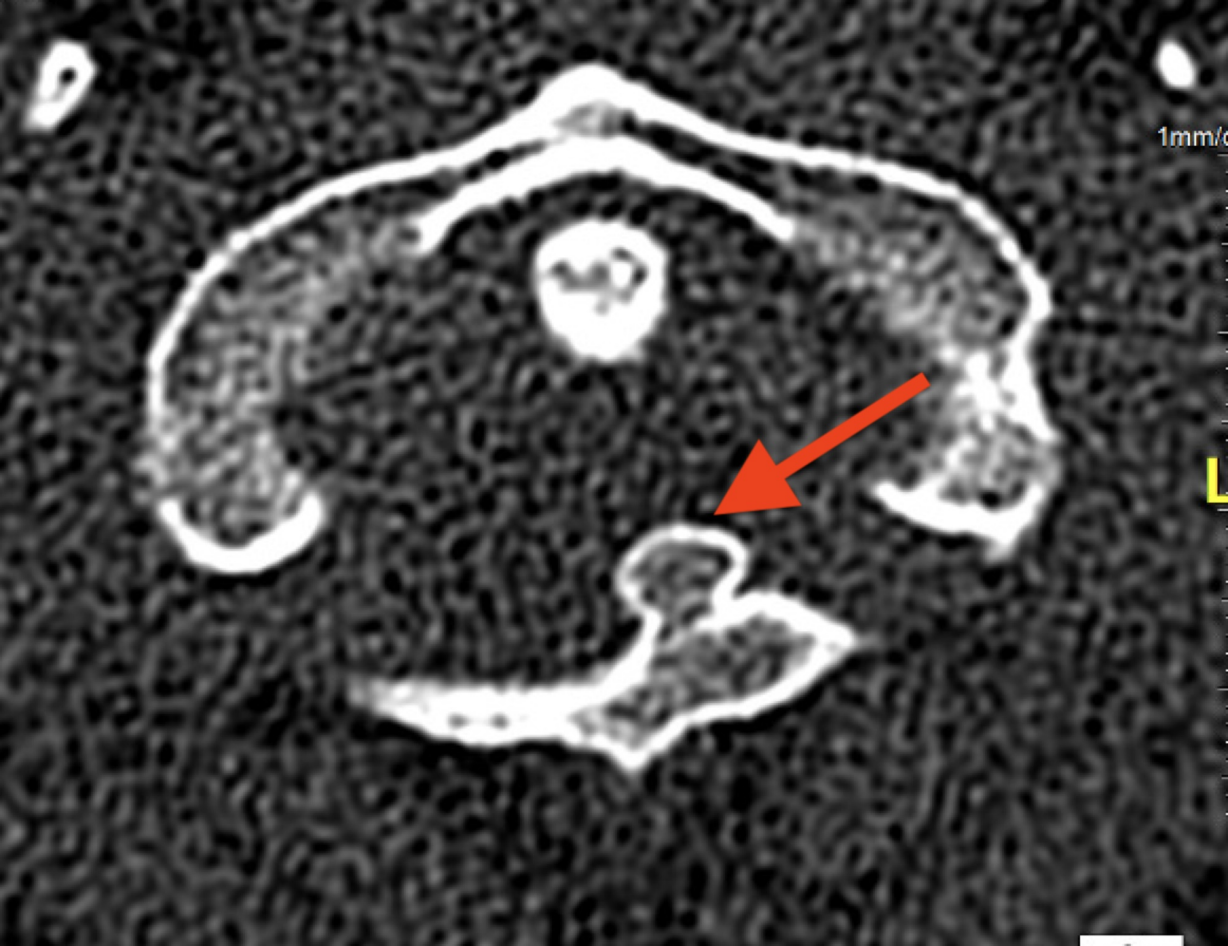
Cervical CT axial view demonstrating a pedunculated osteochondroma on the posterior arch of C1

**Figure 4:**
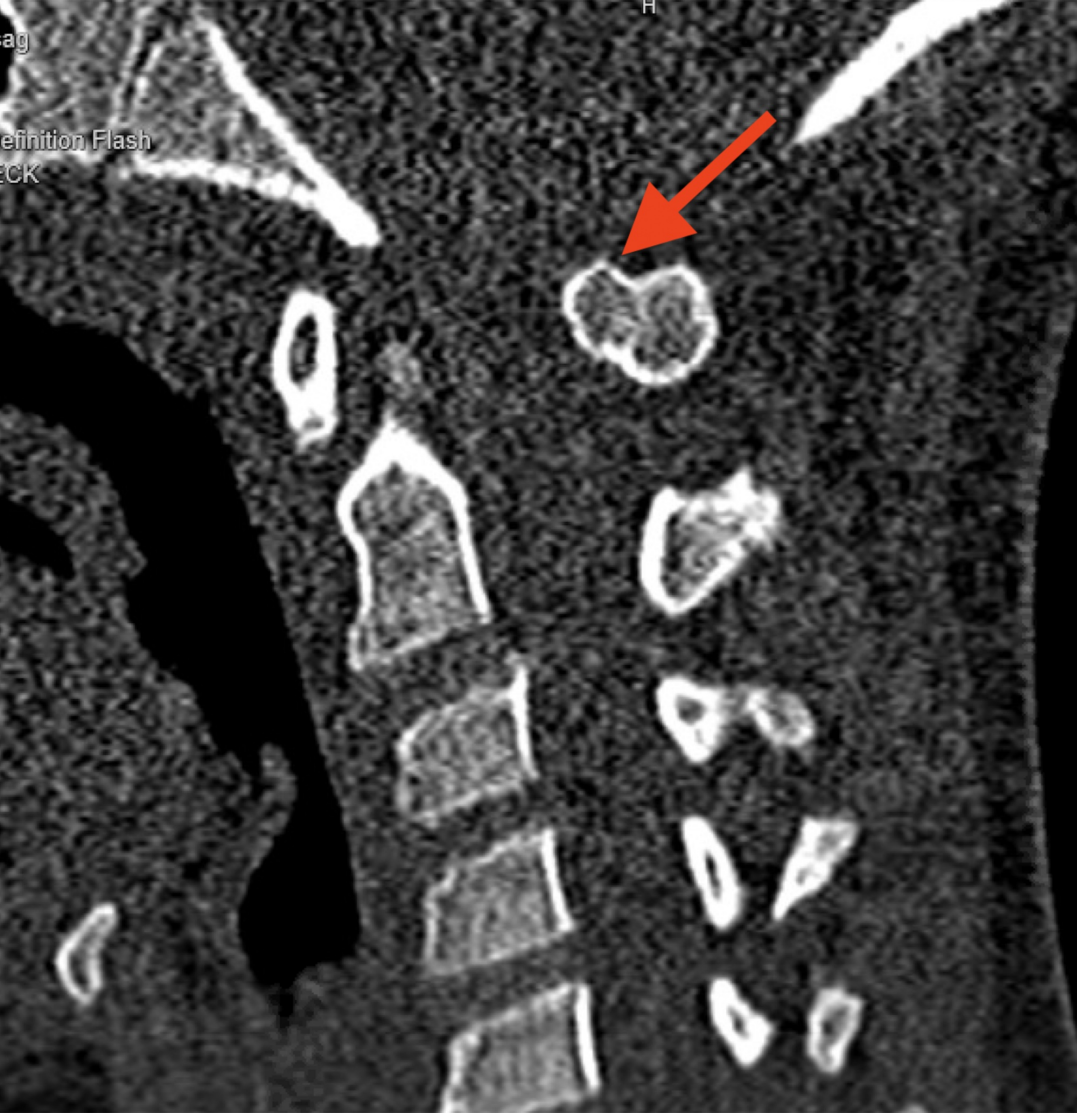
Cervical CT sagittal view demonstrating a pedunculated osteochondroma on the posterior arch of C1

Screening spinal MRI was offered to the family prior to planned forearm surgery, and they elected to proceed with the MRI before the forearm surgery. The MRI identified a C1 osteochondroma originating from the posterior arch and compressing the spinal cord, as well as multiple osteochondromas on the right-sided facets at T6 and L3 (Figures 1-4).

Activity restrictions were placed, and she was referred to a spine surgeon. She underwent elective C1 laminectomy soon thereafter, had an uneventful recovery, and proceeded with reconstructive forearm surgery. She maintained full neurologic function throughout the entire clinical course.

## Results

**Figure 5:**
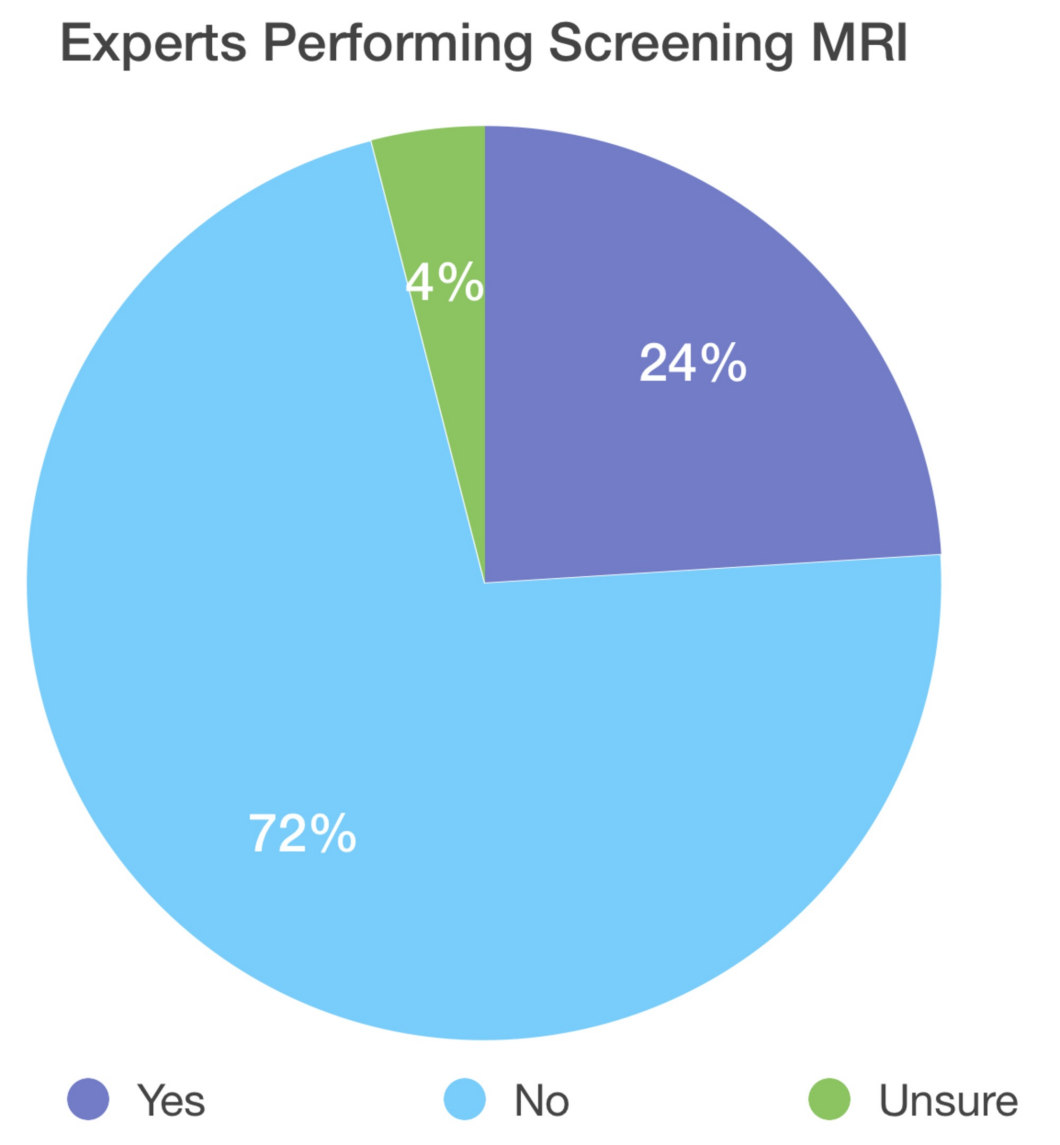
Display of the percentage of experts who are currently performing a routine screening spinal MRI in asymptomatic patients with MHE; 72% of experts are not performing screening MRI MHE: multiple hereditary exostoses

**Figure 6:**
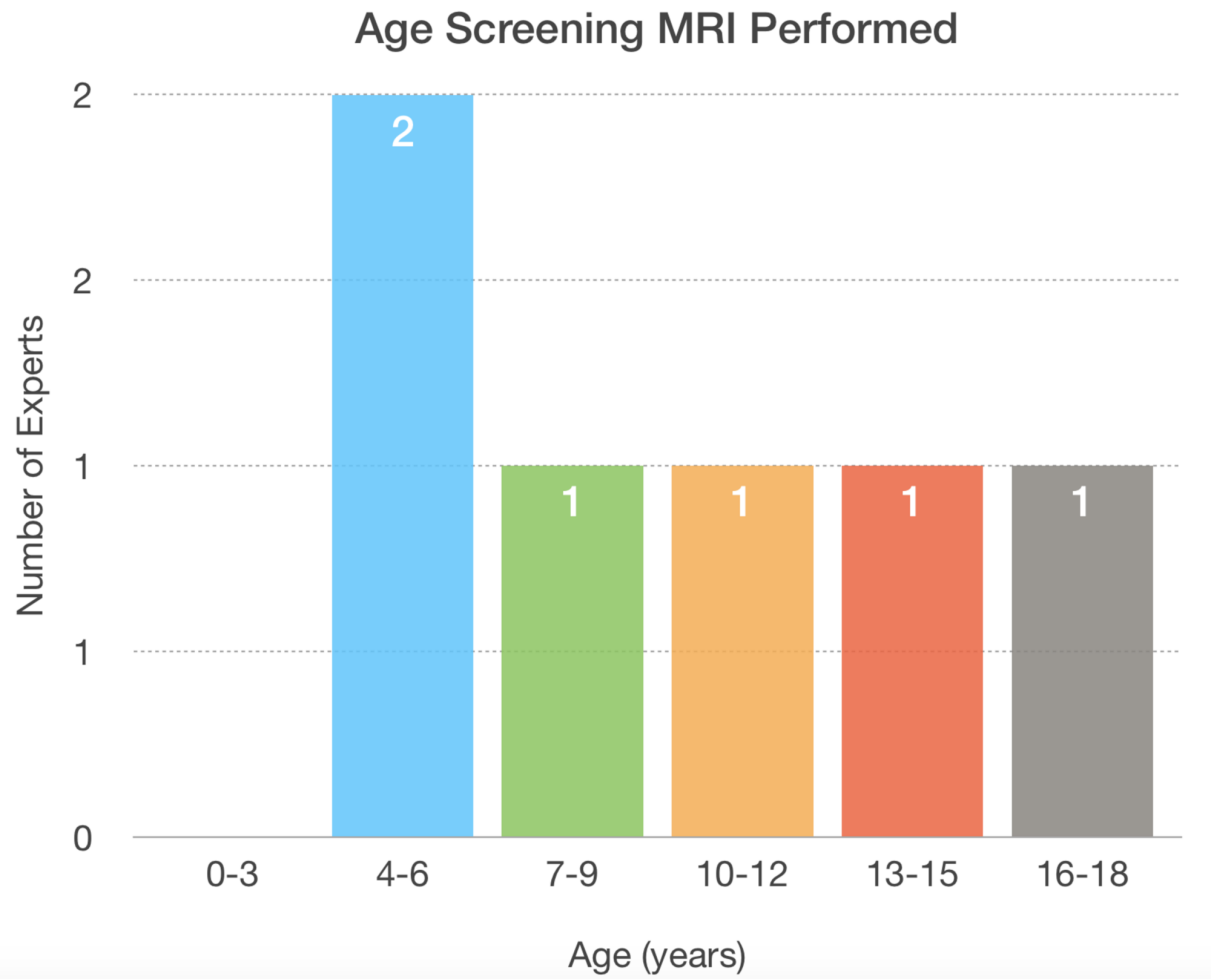
The age range to screen the spine is widely distributed for experts that are performing screening spinal MRI

A total of 104 experts were contacted, with a total of 26 experts participating in the survey and 22 completing the entire survey. Of the respondents, 77.3% (17 of 22) work at academic centers while 38.4% (10 of 26) of respondents work at high-volume centers that are currently following 51 or more pediatric patients with MHE, 23.1% (6 of 26) are currently following between 26-50 patients with MHE, 7.7% (2 of 26) are following between 11 and 25 patients with MHE, and 30.7% are following less than 10 patients with MHE (8 of 26). Seventy-two percent (18 of 25) of respondents do not perform routine MRI screening of the spine, 24.0% (6 of 25) of the respondents perform routine MRI of the spine, and one respondent was unsure if routine MRI is performed at their institution (Figures 5-6).

For experts that routinely screen, screening is performed across a wide age range, for patients anywhere between four and 18 years old. When asymptomatic intracanal spinal lesions that indent the dura are discovered 47.8% (11 of 23) consider removing the lesion based on the size and location, 30.4% (7 of 23) of respondents do not resect asymptomatic lesions, and 21.7% (5 of 23) were unsure. When asked about activity restrictions for intracanal asymptomatic spinal lesions that indent the dura 47.8% (11 of 23) of respondents provide activity restrictions depending on the size and location of the lesion, 21.7% (5 of 23) do not provide activity restrictions, 8.7% (2 of 23) routinely provide activity restrictions, and 21.7% (5 of 23) were unsure of the protocol at their institution. When asked if a routine MRI of the spine should be performed, 60.9% (14 of 23) responded “No” and 39.1% (9 of 23) responded “Yes.” For experts who thought a routine MRI should be performed, the ideal age for screening varied widely from four to 20 years old.

## Discussion

As in our case, consensus guidelines are lacking to help physicians and families determine if a spinal screening MRI should be performed for patients with MHE, and, if so, when it should be done. This study aimed to elucidate the current spinal screening MRI practices of MHE experts.

Screening spinal MRI in patients with MHE is controversial. The risk of rapid and permanent neurologic damage is the primary reason to screen an asymptomatic patient [5,13-14]. The optimal timing and frequency of MRI screening is still undetermined [5,10]. After reporting that a high percentage of MHE patients have intraspinal osteochondromas, Roach et al. recommended screening MRI of the entire spinal column on all patients with MHE at least once during the growing years of life. They prefer that screening MRI be performed as soon as the child can cooperate and hold still to avoid general anesthesia. Reasons authors have chosen not to recommend screening include an unclear rate of intracanal involvement, favorable neurologic recovery if symptomatic lesions are resected soon after symptom onset, the expense of the MRI, and the side effects of sedation [4,13,15-19]. Additionally, those against routine screening have voiced concerns regarding resource utilization and potential overdiagnosis based on incidental findings following imaging [4]. Another confounding factor is that intracanal lesions identified on imaging are commonly asymptomatic and imaging is poorly predictive of their rate of progression [5,18].

The lack of clarity has prompted research into selectively screening patients. Patients may be at a higher risk for spine osteochondromas if they have pelvic and rib osteochondromas or “harbinger” lesions [4]. Jackson et al. reported 100% sensitivity and 69% specificity when using harbinger lesions to identify spine osteochondromas. In order to appropriately stratify patients using this method, imaging of the chest and pelvis would be required. The patient from our case report had pelvic osteochondromas but imaging of her ribs was not obtained and, therefore, the presence of rib osteochondromas was unknown.

The risks of acute severe neurologic damage with long-term sequelae or death in the previously asymptomatic patient are exceedingly rare [5,9,20]. Roach et al. described a 14-year-old boy with MHE and two intracanal lesions who developed acute quadriplegia after minor trauma with long-term sequelae [5]. Spinal screening may have improved the outcome. Roach et al. also reported that a second patient experienced neurological symptoms for one year and had complete paraplegia before a thoracic lesion was identified. Perhaps MRI screening would have allowed for closer monitoring or even removal prior to deleterious neurological effects. There are many other reports of patients who had acute neurologic deterioration, however, the outcome is overwhelmingly favorable after prompt resection, with most patients returning to baseline function [8,13,16-17,21-22].

While we solicited opinions on clinical practice from experts based on the publicly available literature, the practice patterns of these experts are markedly variable. Variability was noted for the nearly four-in-10 experts practicing at high volume centers. Nearly three-quarters of experts do not currently perform routine spinal screening MRI. When screening MRI is performed, the age at which it is performed is ranged from four years old to 18. Wide variation also exists with treatment recommendations and activity restriction for patients with asymptomatic intracanal spinal osteochondromas. Greater than one in five experts were unsure of the next steps in management following a scan positive for intracanal spinal lesions indenting the dura.

This study has limitations. The first is the relatively low response rate to the survey. While the response rate was low, we believe 26 experts participating allows for insight into current practice trends. An additional inherent limitation of our survey study is that it depends on respondents to accurately report practice decisions and imaging orders.

## Conclusions

This survey clearly demonstrates a lack of consensus for a clinical question that healthcare providers have battled for years: should asymptomatic patients with MHE have routine spinal screening? Currently, healthcare providers caring for patients with MHE are left to decipher a conflicting body of evidence, which has led to great variability in practice patterns. Requesting a spinal MRI for a child is expensive and can be disruptive to the patient and family if not necessary. In order to reconcile the wide variability in the treatment of these patients, there is a need to establish clear guidelines to determine if, and when, screening MRI is necessary for patients with this rare disease. Further investigation is also necessary to determine if “harbinger” lesions can be used to selectively screen patients and to verify if “harbinger” lesions improve sensitivity as much as previously reported.

## Appendices

1. How many patients with multiple hereditary exostoses under the age of 18 are currently being followed by your center?

a. 0-10

b. 11-25

c. 26-50

d. 51 or more

2. Which category accurately describes your workplace?

a. Academic

b. Non-academic

3. Does your center routinely perform an MRI screen of the spine in patients with MHE?

a. Yes (if yes free text age)

b. No

4. Does your center routinely provide activity restrictions for asymptomatic intracanal spinal lesions that indent the dura?

a. Yes

b. No

c. Not routinely, but may provide restrictions depending on the size of the osteochondroma and/or level of the osteochondroma

d. Unsure

5. Does your center routinely remove asymptomatic intracanal osteochondromas that indent the dura?

a. Yes

b. No

c. Not routinely, but may remove the osteochondroma depending on the size and/or level of the osteochondroma

d. Unsure

6. Have you or someone in your practice followed a patient with MHE that had a permanent neurologic deficit from an intracanal osteochondroma?

a. Yes

b. No

c. Unsure

7. Do you think a routine MRI screen of the spine should be recommended for all patients with MHE?

a. Yes (if yes ask to free text age)

b. No

8. Do you think activity restrictions should be recommended for all patients with osteochondromas indenting the dura?

a. Yes

b. No

c. Not routinely, but provide restrictions depending on size of osteochondroma and level of the osteochondroma

9. Do you think asymptomatic osteochondromas should be removed?

a. Yes

b. No

c. Not routinely, but sometimes the osteochondroma should be removed depending on size and level of the osteochondroma
